# Algorithms for joint activity–attenuation estimation from positron emission tomography scatter

**DOI:** 10.1186/s40658-019-0254-y

**Published:** 2019-10-28

**Authors:** Yannick Berker, Volkmar Schulz, Joel S. Karp

**Affiliations:** 1 Division of X-ray Imaging and Computed Tomography, German Cancer Research Center (DKFZ), Im Neuenheimer Feld 280, Heidelberg, 69120 Germany; 2 Department of Physics of Molecular Imaging Systems, Institute for Experimental Molecular Imaging, RWTH Aachen University, Forckenbeckstraße 55, Aachen, 52074 Germany; 3 Department of Radiology, University of Pennsylvania, 3620 Hamilton Walk, Philadelphia, 19104 PA USA; 4 III. Physikalisches Institut B, RWTH Aachen University, Otto-Blumenthal-Straße, Aachen, Germany; 5 Fraunhofer Institute for Digital Medicine MEVIS, Forckenbeckstraße 55, Aachen, 52074 Germany

**Keywords:** Positron emission tomography, Image reconstruction, Attenuation correction, Compton scattering

## Abstract

**Background:**

Attenuation correction in positron emission tomography remains challenging in the absence of measured transmission data. Scattered emission data may contribute missing information, but quantitative scatter-to-attenuation (S2A) reconstruction needs to input the reconstructed activity image. Here, we study S2A reconstruction as a building block for joint estimation of activity and attenuation.

**Methods:**

We study two S2A reconstruction algorithms, maximum-likelihood expectation maximization (MLEM) with one-step-late attenuation (MLEM-OSL) and a maximum-likelihood gradient ascent (MLGA). We study theoretical properties of these algorithms with a focus on convergence and convergence speed and compare convergence speeds and the impact of object size in simulations using different spatial scale factors. Then, we propose joint estimation of activity and attenuation from scattered and nonscattered (true) emission data, combining MLEM-OSL or MLGA with scatter-MLEM as well as trues-MLEM and the maximum-likelihood transmission (MLTR) algorithm.

**Results:**

Shortcomings of MLEM-OSL inhibit convergence to the true solution with high attenuation; these shortcomings are related to the linearization of a nonlinear measurement equation and can be linked to a new numerical criterion allowing geometrical interpretations in terms of low and high attenuation. Comparisons using simulated data confirm that while MLGA converges largely independent of the attenuation scale, MLEM-OSL converges if low-attenuation data dominate, but not with high attenuation. Convergence of MLEM-OSL can be improved by isolating data satisfying the aforementioned low-attenuation criterion. In joint estimation of activity and attenuation, scattered data helps avoid local minima that nonscattered data alone cannot. Combining MLEM-OSL with trues-MLEM may be sufficient for low-attenuation objects, while MLGA, scatter-MLEM, and MLTR may additionally be needed with higher attenuation.

**Conclusions:**

The performance of S2A algorithms depends on spatial scales. MLGA provides lower computational complexity and convergence in more diverse setups than MLEM-OSL. Finally, scattered data may provide additional information to joint estimation of activity and attenuation through S2A reconstruction.

## Introduction

Positron emission tomography (PET) is an important noninvasive medical imaging modality for clinical and research applications [1], with particular strengths in sensitive detection of photon pairs emitted by a radiotracer and quantitative reconstruction of the radiotracer activity image *λ*. PET image reconstruction is usually based on linear models, involving the Radon transform $\mathcal {R} \lambda $ in the analytic case or discrete mappings of a vector $\vec {\lambda }$ in the numerical case, respectively.

For quantitative reconstruction of the activity image, attenuation correction (AC) is essential, compensating for a lack of detected photon pairs along lines of response (LORs) due to the photoelectric effect and Compton scattering in the patient. A complementary step, scatter correction (SC), computes an estimate of extraneous photon pairs along *broken* LORs, which are generated through Compton scattering. Both corrections usually input the spatial distribution of the electron density *ρ* in the form of a map of linear attenuation coefficients *μ* or, for AC purposes, the so-called attenuation *sinogram*$\mathcal {R} \mu $. Given *μ*, both AC and SC are state of the art using well-validated algorithms [2, 3], but vast research efforts had to be—and still are—directed to the determination of *μ*.

### Determination of an attenuation map

Depending on the level of integration of PET with other modalities (standalone or multi-modality PET), information from radionuclide transmission sources [4, 5], X-ray computed tomography (CT) [6], or magnetic resonance imaging (MRI) [7] can be used. However, radionuclide transmission data suffer from low signal-to-noise ratio, necessitating segmentation to prevent noise in the transmission data from impacting activity images. In PET/CT, 4-D attenuation correction of PET data acquired from a moving subject remains limited due to concerns over radiation doses induced by cine CT imaging. In PET/MRI outside of the head/neck area, MRI is often incapable of distinguishing bone from air in reasonable scan times [8].

More universal approaches to determine *μ* do not depend on multi-modality information. Popularized through maximum-likelihood reconstruction of attenuation and activity (MLAA, [9–11]), these algorithms use only PET emission data, replacing the optimization problem in *λ* by a joint problem in (*λ*,*μ*). A recently proposed group of algorithms jointly estimate the activity image and the attenuation sinogram $\mathcal {R} \mu $, either using alternation [12, 13] or simultaneous updates [14].

Time-of-flight (TOF) PET emission data determine the attenuation sinogram $\mathcal {R} \mu $, but only on LORs with activity ($\mathcal {R}\lambda > 0$) and only up to an unknown offset [15]. The former limitation is not a severe issue for AC, where other values of $\mathcal {R} \mu $ are not needed. However, it complicates reconstruction of *μ* from $\mathcal {R} \mu $ and therefore is a problem for SC, where an image-space *μ*-map is usually required. The latter limitation translates into an unknown scaling factor in the reconstructed *λ*. For these reasons, AC and SC using only PET emission data are still impractical [16].

Another type of available data is low-energy, object-scattered PET emission data, which may contain enough additional information to address both aforementioned limitations [17]: particularly, in a joint reconstruction scheme [18]. Similar opportunities arise in single-photon emission computed tomography [19–22]. Unfortunately, the model of the measured PET scatter data is neither based on the regular Radon transform nor linear in *μ*. A maximum-likelihood gradient ascent algorithm for scatter-to-attenuation (S2A) reconstruction has therefore been proposed [23, 24] but, so far, not been used in joint estimation. Most recently, a Broyden–Fletcher–Goldfarb–Shanno (BFGS)-based algorithm has been proposed for attenuation reconstruction from coincidences in a lower energy window [25, 26].

The problem of estimating attenuation from scattered PET photons shares similarities with Compton scatter imaging, in which external Gamma sources are used to probe an object’s electron density for medical [27] or industrial [28] applications. While it is known from the latter that the nonlinearity of the problem favors thin, low-density objects, the impact of object size in scatter-based PET attenuation correction remains to be studied.

### Objectives

This paper is thus concerned with characterizing S2A reconstruction as a building block in joint estimation of activity and attenuation (*joint estimation*). We follow three objectives: (1) further understand fundamental properties impacting convergence and convergence speed of S2A algorithms; (2) compare S2A algorithms using simulated data, specifically, in terms of convergence speed, the impact of object size, and improved performance of one algorithm by reducing its input data; and (3) study joint estimation, which implies dropping the assumption of known radiotracer activity images in S2A reconstruction [17, 24]. Therefore, we integrate scatter data into joint estimation by interleaving S2A reconstruction with trues-to-activity reconstruction, as proposed before [18, 29], as well as with trues-to-attenuation and scatter-to-activity reconstruction.

In this algorithmically oriented proof of concept, studies are carried out using 2-D digital phantoms and simulations restricted to single scattering without TOF information. Furthermore, we assume perfect energy resolution that enables ideal separation of scattered and nonscattered events and noise-free data.

After statement of the problem, introducing required imaging models for use in S2A reconstruction and joint estimation, we summarize and propose algorithms for both and describe the evaluation data used and the experiments carried out, before presenting and discussing our results.

## Problem statement

This section summarizes notation and models for scattered and unscattered data.

### Scattered data for S2A reconstruction

Scatter-to-attenuation reconstruction requires a model of the low-energy, scattered data. Therefore, we identify the coincident detection of two photons along an LOR by the involved detector pair. If exactly one of two detected photons has been object-scattered exactly once, the coincidence is said to be single-scattered and the energy of that photon is denoted *E*. We denote the respective detector *d*_*s*_ (*scattered*) and the other *d*_*n*_ (*nonscattered*). Thus, a tuple *i*=(*d*_*s*_,*d*_*n*_,*E*) comprises all properties of a single-scattered coincidence used in this work; *l*=(*d*_*s*_,*d*_*n*_) denotes a regular LOR.

**Fig. 1:**
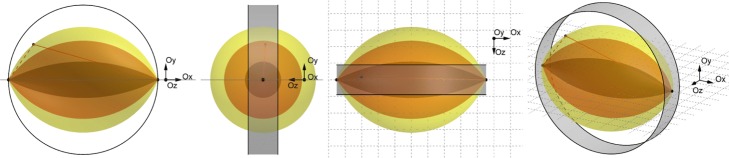
Front, side, top, and oblique views of SORs for coincidences detected in opposite detector elements on a detector surface (gray) for three different energies of the scattered photon: 460 keV (innermost, darkest), 358 keV (middle), and 307 keV (outermost, lightest). Assuming the scattered photon in the left detector, one potential broken LOR is indicated, with the solid part indicating potential activity source locations. Each broken LOR runs inside its SOR, touching it only at the detectors and one potential scattering location. Adapted, with permission, from ([17], Figure 2). Ⓒ 2014 American Association of Physicists in Medicine

The trajectory of both photons is a broken LOR as shown in Fig. 1, connecting a scattering location $\vec {x}_{s}$ with both detector locations. Unfortunately, many different broken LORs, in particular, having different scattering locations, yield the same apparent LOR *l*, so that the photon trajectory, and in particular, the scattering location, cannot be determined from *l*. It is known, however, that the true $\vec {x}_{s}$ lies on an American-football-shaped surface of revolution, with the pointed ends in the detector locations and the radius determined by *E*. We use *i* to denote this *surface of response* (SOR), comprising all possible scattering locations for a single-scattered coincidence^1^. For each scattering location $\vec {x}_{s}$ in SOR *i*,(*i*,*s*) describes one potential broken LOR.

In list-mode acquisition, the raw scatter data is a sequence (*i*_1_,*i*_2_,…); after histogramming, the data is the number of detected single-scattered coincidences for each possible *i*. Here, *y*^*i*^ denotes the simulated or measured data on SOR *i*, while $\bar {y}^{{i}}$ is used for the expected data. The dimension of the data space is $N_{{i}} \leq N_{d}^{2} \times N_{E}$, with *N*_*d*_ detectors and *N*_*E*_ energy bins (or equivalently, energy windows)^2^.

Voxels are indexed according to their physical roles using *e* (*emitting*), *s* (*scattering*), and *t* (*transmitting*). A 2-D matrix ***A***_*λ*_ (with entries $a^{{i}}_{s}$) describes the sensitivity of the PET camera on SOR *i* for radiation scattered in a voxel *s* in the absence of attenuation; it integrates both normalized camera sensitivity (scatter geometry, photon detection efficiency) and the object’s source density *λ*, as detailed in the Appendix. A 3-D tensor $\underline {\boldsymbol {K}}$ (with entries $k^{{i}}_{s,t}$) represents the attenuating path length of that radiation through a voxel *t*, independent of the object.

The expected number of low-energy scatter coincidences $\vec {\bar {y}}$, which is linear in the activity $\vec {\lambda }$, is modeled according to a discretized variant of the scatter-measurement equation (23), a generalization of the single scatter simulation (SSS) equation [3]. Using the notation in Table 1, we write the discrete measurement equation as:

1 $$ \bar{y}^{{i}} = \sum_{s}\left(\sum_{e} b^{{i}}_{s,e} \lambda^{e}\right) \exp \left(- \sum_{t} k^{{i}}_{s,t} \rho^{t}\right) \rho^{s} = \sum_{s} a^{{i}}_{s} \exp \left(- \sum_{t} k^{{i}}_{s,t} \rho^{t}\right) \rho^{s}  $$

**Table 1:** Notation used for mathematics, image space, measurement space, and physics

Symbol	Description
$\underline {\boldsymbol {T}}, \boldsymbol {M}, \vec {v}, \vec {1}_{[\cdot ]}$	Rank-3 tensor, matrix, vector, vector composed of ones
⊗; $\odot, \oslash, \overset {\circ }{\text {exp}}$	Outer product; element-wise multiplication, division, exp,
$\vec {\lambda }, \vec {\mu }, \vec {\rho }$	Spatial distribution of activity, linear attenuation coefficient, electron density
*j*, *e*, *s*, *t*	Indices of all, emitting, scattering, transmitting voxels
$\vec {y}, \vec {\bar {y}}$; $\vec {z}, \vec {\bar {z}}$	Measured/simulated, expected scatter (*y*) or trues (*z*) data
*d*_*s*_,*d*_*n*_	Detectors of scattered, nonscattered photons of a single-scatter coincidence
*E*, *θ*	Energy of scattered photon, associated scattering angle
*l*	=(*d*_*s*_,*d*_*n*_), index of an LOR
*i*	=(*d*_*s*_,*d*_*n*_,*E*), index of an SOR
(*i*,*s*)	=(*d*_*s*_,*d*_*n*_,*E*,*s*), index of a broken LOR
*N*_*d*_,*N*_*E*_,*N*_*l*_,*N*_*i*_	Numbers of detectors, energy bins, LORs, SORs
$\boldsymbol {A}, \boldsymbol {A}_{\omega }, \boldsymbol {\tilde {A}}$	SOR system matrices: (***A***_*ω*_, function of parameters;${~}^{\mathrm {a}} \boldsymbol {\tilde {A}}$ with attenuation)
$\boldsymbol {U}, \boldsymbol {\tilde {U}}_{\rho }$	LOR system matrices (as above)
$\underline {\boldsymbol {B}}, b^{{i}}_{s,e}$	Probability that radiation emitted in *e* is detected along the broken LOR (*i*,*s*), disregarding attenuation, per unit electron density in *s*, per unit activity in *e*
$\underline {\boldsymbol {{L}}}, l^{{i}}_{s,t}$	Effective intersection length of photon path along the broken LOR (*i*,*s*) with the transmitting (or attenuating) voxel *t*, taking photon energy into account
$\underline {\boldsymbol {{K}}}, k^{{i}}_{s,t}$	=*ls*,*ti*·*μ*^*t*^/*ρ*^*t*^, effective intersection length times LAC–electron-density ratio
$\mathcal {L}_{y}(\bullet)$	Poisson log-likelihood (LL) given the measured data $\vec {y}$
$\mathcal {S}(\bullet, \vec {x}^{\,\text {true}})$	Normalized mean squared error (NMSE) with respect to a reference $\vec {x}^{\text {true}}$

^a^These parameters are assumed to be constant within one iteration, but can be updated between iterations

or, in matrix notation, denoting the element-wise operations by ⊙ and $\overset {\circ }{\text {exp}}$:

2 $$ \vec{\bar{y}} (\vec{\lambda}, \vec{\rho}) = \left(\boldsymbol{A}_{\lambda} \odot \overset{\circ}{\text{exp}} (- \underline{\boldsymbol{K}} \vec{\rho})\right) \vec{\rho}.  $$

The above expressions are equivalent to (41) and (42), respectively. Their derivation is subject to the following assumptions:

*ks*,*ti*,*e*=*ks*,*ti* Effective attenuation lengths of a voxel *t* seen by photons along a broken LOR (*i*,*s*) are independent of the point of emission *e* along that broken LOR: this is a common assumption in PET that has been fundamental in showing that TOF PET data determine the attenuation sinogram up to a constant [15].

*μ*∝*ρ* The linear attenuation coefficient *μ* is proportional to the electron density *ρ*: approximately, this is true because in biological (low-Z) materials at PET energies, Compton scattering is the dominant interaction preventing gamma photon pairs from being detected. At fixed energy, the ratio *μ*/*ρ* can be formulated to depend on the mass attenuation coefficient (*μ*/*ρ*_*m*_) and the quotient of mass density and electron density (*ρ*_*m*_/*ρ*). The former is fairly constant across human tissues at PET energies ([30], Fig. 3), and the latter is almost perfectly constant for materials less dense than water and deviates a maximum of 10% for materials three times as dense ([31], Fig. 1). Note that *μ*/*ρ* may well depend on the photon energy; no assumption about the energy dependence of *μ* is implied (see the discussion around (35)).

If we assume that the electron density $\vec {\rho }$ is known accurately enough to approximate attenuation effects, as we will for one algorithm, we can simplify (2) further. That is, with an estimate $\vec {\rho }^{\,\text {est}}$ and using the abbreviation $\boldsymbol {\tilde {A}}_{\lambda,\rho } := \boldsymbol {A}_{\lambda } \odot \overset {\circ }{\text {exp}} (- \underline {\boldsymbol {K}} \vec {\rho })$, we find the linear mapping:

3 $$  \vec{\bar{y}}' = \boldsymbol{\tilde{A}}_{\lambda,\rho^{\,\text{est}}} \vec{\rho} \iff \bar{y}'^{{i}} = \sum_{{j}} \tilde{a}^{{i}}_{{j}} \rho^{{j}}.  $$

We denote attenuated system matrices by a tilde (see Table 1; $\tilde {a}$ for components) and refer to (3) as the linearization of the scatter measurement equation.

### Unscattered data for joint estimation

Our joint estimation approach requires, additionally, a model of the nonscattered data and three well-known algorithms. We assume the LOR model with:

4 $$ \begin{aligned}  \vec{\bar{z}} &= (\boldsymbol{U} \vec{\lambda}) \odot \exp (- \boldsymbol{U} \vec{\rho})= \boldsymbol{\tilde{U}}_{\rho} \vec{\lambda}\\ &\Longleftrightarrow \quad \bar{z}^{{l}} = \left(\sum_{{j}} u^{l}_{j} \lambda^{j}\right) \exp \left(- \sum_{j} u^{l}_{j} \rho^{j}\right) = \sum_{j} \tilde{u}^{l}_{j} \lambda^{j}, \end{aligned}  $$

where $\vec {\bar {z}}$ is the expected nonscattered data, ***U*** is the LOR system matrix without attenuation, i.e., the usual system matrix applied for the usual PET reconstruction (see Appendix), and $\boldsymbol {\tilde {U}}_{\rho }$ the attenuated one; $\smash {u^{{l}}_{{j}}}$ and $\smash {\tilde {u}^{{l}}_{{j}}}$ represent entries of ***U*** and $\boldsymbol {\tilde {U}}_{\rho }$, respectively, for LOR *l* and voxel $\smash {{j}}$.

These nonscattered (true) data are used by maximum-likelihood expectation-maximization [32], which we refer to as trues-MLEM:

5 $$  \vec{\lambda}^{\text{new}} = \vec{\lambda} \odot \left(\boldsymbol{\tilde{U}}_{\rho}^{{\top}} \left(\vec{z} \oslash \vec{\bar{z}}\right)\right) \oslash \left(\boldsymbol{\tilde{U}}_{\rho}^{{\top}} \vec{1}_{[{l}]}\right),  $$

and by the relaxed maximum-likelihood transmission algorithm [33] (trues-MLTR):

6 $$  \vec{\rho}^{\text{new}} = \vec{\rho} + \eta \cdot \left(1 - \left(\boldsymbol{U}^{\top} \vec{z}\right) \oslash \left(\boldsymbol{U}^{{\top}} \vec{\bar{z}}\right)\right).  $$

In addition, scatter-MLEM [34] will be used, of which a brief derivation is given in the Appendix. This algorithm’s update equation reads:

7 $$  \vec{\lambda}^{\text{new}} = \vec{\lambda} \odot \left(\boldsymbol{\tilde{A}}_{\rho}^{\top} \left(\vec{y} \oslash \vec{\bar{y}}\right)\right) \oslash \left(\boldsymbol{\tilde{A}}_{\rho}^{\top} \vec{1}_{[{i}]}\right).  $$

## Methods and materials

In this section, we summarize two recent S2A algorithms and then look at fundamental differences between them. We then propose two novel joint estimation approaches using these algorithms and present our evaluation strategy.

### Gradient-based algorithms for S2A reconstruction

In previous work, we introduced the two-branch back-projection (2BP) algorithm [17] which chooses between a positive and a negative update of *ρ* in a binary random-walk fashion. Since we found this algorithm to be impractical for most applications [24], we focus on two gradient-ascent-based algorithms here.

The Poisson log-likelihood (LL) of some expected data $\vec {\bar {y}}$, given the data $\vec {y}$ and omitting terms that do not depend on $\vec {\bar {y}}$, reads:

8 $$  \mathcal{L}_{y}(\vec{\bar{y}}) = \sum_{{i}} \left(y^{{i}} \log \bar{y}^{{i}} - \bar{y}^{{i}}\right),  $$

with its gradient with respect to a vector $\vec {\rho }$

9 $$  \vec{\nabla}_{\vec{\rho}} \mathcal{L}_{y} = \left(\vec{\nabla}_{\vec{\rho}} \otimes \vec{\bar{y}}\right) \left(\vec{y} \oslash \vec{\bar{y}} - \vec{1}_{[{i}]}\right) \quad\iff\quad \frac{\partial \mathcal{L}_{y}}{\partial \rho^{{j}}} = \sum_{{i}} \frac{\partial \bar{y}^{{i}}}{\partial \rho^{{j}}} \left(\frac{y^{{i}}}{\bar{y}^{{i}}}- 1\right).  $$

For the linearization (3), since $\boldsymbol {\tilde {A}}_{\lambda,\rho ^{\,\text {est}}}$ does not depend on $\vec {\rho }$, we find the gradient of the expected data to be:

10 $$  \vec{\nabla}_{\vec{\rho}} \otimes \vec{\bar{y}} = \boldsymbol{\tilde{A}}_{\lambda,\rho^{\,\text{est}}}^{{\top}} \quad\iff\quad \frac{\partial \bar{y}^{{i}}}{\partial \rho^{{j}}} = \tilde{a}^{{i}}_{{j}}.  $$

By contrast, observing the double dependence of (2) on $\vec {\rho }$, one finds:

11a $$\begin{array}{*{20}l} \vec{\nabla}_{\vec{\rho}} \otimes \vec{\bar{y}} &= \left[\boldsymbol{A}_{\lambda}\odot\overset{\circ}{\text{exp}}(- \underline{\boldsymbol{K}}\vec{\rho})-\vec{\rho}^{\top} \left\{\underline{\boldsymbol{K}}\odot\left(\left[\boldsymbol{A}_{\lambda}\odot\overset{\circ}{\text{exp}}(-\underline{\boldsymbol{K}}\vec{\rho}) \right]\otimes\vec{1}_{[t]}\right)\right\}\right]^{\top} \end{array} $$

11b $$\begin{array}{*{20}l}  &= \left[\boldsymbol{\tilde{A}}_{\lambda,\rho} - \vec{\rho}^{\top} \left\{\underline{\boldsymbol{K}}\odot\left(\boldsymbol{\tilde{A}}_{\lambda,\rho}\otimes\vec{1}_{[t]}\right)\right\}\right]^{\top} \end{array} $$

instead, which simplifies to (10) only under $\vec {\rho } = 0$ or $\underline {\boldsymbol {K}}=0$ (nonscattering or nonattenuating object) and $\vec {\rho }^{\,\text {est}} = \vec {\rho }$. This vectorial expression lends itself particularly well to an implementation in MATLAB (The MathWorks, Natick, MA); note that the multiplication with $\vec {\rho }^{{\top }}$ (from the left) denotes a summation over the scattering voxels *s*, as indicated in the component-wise expression:

12a $$\begin{array}{*{20}l} \frac{\partial \bar{y}^{i}}{\partial \rho^{j}} & =a^{i}_{j} \exp \left(- \sum_{t} k^{i}_{{j},t} \rho^{t}\right) -\sum_{s} \rho^{s} k^{i}_{s,{j}} a^{i}_{s} \exp \left(- \sum_{t} k^{i}_{s,t} \rho^{t}\right) \end{array} $$

12b $$\begin{array}{*{20}l}  & = \tilde{a}^{i}_{j} - \sum_{s} \rho^{s} k^{i}_{s,{j}} \tilde{a}^{{i}}_{s}. \end{array} $$

#### Scatter-to-attenuation MLEM with one-step-late attenuation (MLEM-OSL)

This algorithm, called MLEM by its authors [18], is based on subsuming attenuation effects under the system matrix in the linearized measurement equation (3), yielding the MLEM update [32]:

13 $$ \vec{\rho}^{\text{new}} = \vec{\rho}\odot\left(\boldsymbol{\tilde{A}}_{\lambda,\rho}^{\top}\left(\vec{y} \oslash \vec{\bar{y}}\right)\right) \oslash \left(\boldsymbol{\tilde{A}}_{\lambda,\rho}^{\top}\vec{1}_{[{i}]}\right) \quad\Longleftrightarrow\quad \rho^{{\text{new}},{j}} = \rho^{j} \frac{\sum_{i} \tilde{a}^{i}_{j} {y^{i}} / {\bar{y}^{i}}}{\sum_{i} \tilde{a}^{i}_{j}}.  $$

However, this update ignores the fact that $\boldsymbol {\tilde {A}}_{\lambda,\rho }$ depends on $\vec {\rho }$, and $\boldsymbol {\tilde {A}}_{\lambda,\rho }$ has to be updated *after* every iteration: Eq. (13) follows the spirit of the so-called one-step-late (OSL) algorithms [35], and we will refer to it as MLEM-OSL here.

#### Maximum-likelihood gradient ascent (MLGA)

The MLEM(-OSL) update (13) can be written as a scaled gradient ascent, with the gradient given by Eqs. (9 and 10) and a vector-valued step size [36]:

14a $$\begin{array}{*{20}l}{2} \vec{\rho}^{\text{new}} & = \vec{\rho} + \underbrace{\vec{\rho}\oslash\left(\boldsymbol{\tilde{A}}_{\lambda,\rho}^{\top} \vec{1}_{[{i}]}\right)}_{\mathrm{step~size}} \odot \vec{\nabla}_{\vec{\rho}} \mathcal{L}_{y} &\quad\Longleftrightarrow \quad \rho^{{\text{new}},{j}} &= \rho^{j} + \underbrace{\frac{\rho^{j}}{\sum_{i}\tilde{a}^{i}_{j}}}_{\mathrm{step~size}}\frac{\partial \mathcal{L}_{y}}{\partial \rho^{j}} \end{array} $$

14b $$\begin{array}{*{20}l}  & = \vec{\rho} + \vec{s} \odot \vec{\nabla}_{\vec{\rho}} \mathcal{L}_{y} && = \rho^{{j}} + s^{j} \frac{\partial \mathcal{L}_{y}}{\partial \rho^{j}}. \end{array} $$

The closed-form expression of MLGA [23, 24] is obtained from (14b) by inserting the full log-likelihood gradient (9 and 11) and choosing a step size. In this work, we focus on a step size inspired by the MLEM update equation (14a):

15 $$  \vec{s} = \gamma \cdot \vec{\rho} \oslash \left(\boldsymbol{\tilde{A}}_{\lambda,\rho}^{\top}\vec{1}_{[{i}]}\right) \quad\Longleftrightarrow\quad s^{j} = \gamma \frac{\rho^{j}}{\sum_{i} \tilde{a}^{i}_{j}}.  $$

In addition to this MLEM-like step size, two additional step sizes have been tested: the constant step size, proposed before [24], and the scaled nonuniform step size:

16 $$  \vec{s}^{\prime} = \alpha \cdot \vec{1}_{[{j}]} \quad\text{and}\quad \vec{s}^{\prime\prime} = \beta \cdot \vec{\rho}.  $$

Step-size constants *α*,*β*, and *γ* have been optimized empirically for fastest, yet stable convergence with our data.

### Validity of linearizing the scatter measurement equation

The amount of scatter as a function of electron density may not be sufficiently well represented by a linearized measurement equation, and (2) may require more careful treatment. To explore the limits of the linearization, we derive a geometrical interpretation as well as a numerical criterion. This criterion is used to distinguish data that can be used for algorithms based on linearization (here, MLEM-OSL) from data that cannot; further, it is linked to the log-likelihood gradient (11).

**Fig. 2:**
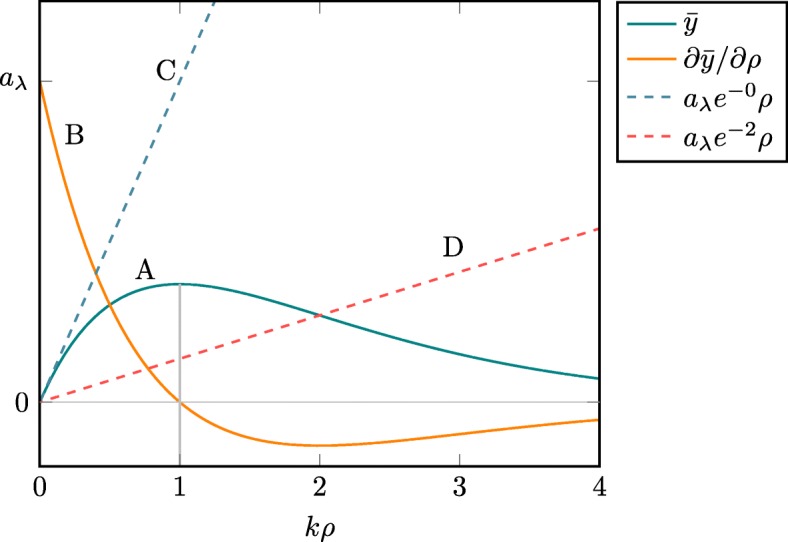
The function $\bar {y} = a_{\lambda } \exp (- k \rho) \rho $ (A), its derivative (B), and linearizations $\bar {y}^{\prime }$ with *kρ*^est^∈{0,2} (C, D)

The most basic, one-dimensional (1-D) simplification of (2):

17 $$ \bar{y}= a_{\lambda} \exp(- k \rho^{\,\text{est}}) \rho \quad (a_{\lambda}, k, \rho^{\,\text{est}}, \rho > 0),  $$

confirms that no possible linearization in the form of (3):

18 $$  \bar{y}' = a_{\lambda,\rho^{\,\text{est}}} \rho,  $$

reflects the behavior of (17) with high attenuation (see Fig. 2): in particular, the derivative of (18) misrepresents the sign of the derivative of (17) for *kρ*>1. This may have drastic implications for gradient-based algorithms using the linearization to compute the gradient: in particular, if *ρ*^*n*^>*ρ*^true^>1/*k*, an iteration of MLEM-OSL yields *ρ*^*n*+1^>*ρ*^*n*^ regardless of the value of *ρ*^est^.

Comparison of the gradient of the linearization (10) with the full gradient (11) reveals the advantage of MLGA over MLEM-OSL; the difference term $- \vec {\rho }^{{\top }} \{\underline {\boldsymbol {K}}\odot [\ldots ]\}$ reverses the direction of the full gradient (only) with high attenuation, all components of $\vec {\rho }$ and $\underline {\boldsymbol {K}}$ being nonnegative^3^.

A multi-voxel interpretation of the high-attenuation situation is presented in the Appendix: it is of importance in patients with great attenuation-length–electron-density products $\vec {\rho }^{\top } \underline {\boldsymbol {K}}$^4^. One conclusion from the arguments in the Appendix is that it is not straightforward to downsample (or downsize) S2A experiments, as that can transform low attenuation into high attenuation (by downsampling), or vice versa (by downsizing)^5^.

For MLEM-OSL, the linearization of the measurement equation may only be appropriate whenever attenuation effects do not reverse the sign of $\partial \bar {y}^{{i}} / \partial \rho ^{{j}}$. Since this may be true for some SORs *i*, but not for others, it may be appropriate to remove the latter from the data and apply MLEM-OSL to the reduced data set: (46) represents an approximate inclusion criterion used later.

### *n*-algorithms for joint estimation

Up to this point, we have focused on dedicated S2A reconstruction algorithms, assuming knowledge of the activity distribution; in this section, we drop this assumption and extend our studies to joint estimation of activity and attenuation using scattered as well as nonscattered data. The added value of combining scattered and nonscattered data is visualized in the Appendix (Fig. 16).

We use five building blocks: the aforementioned MLGA with MLEM-like step size for S2A reconstruction, henceforth referred to as *scatter-MLGA*; *scatter-MLEM-OSL* as an alternative; scatter-MLEM for scatter-to-activity reconstruction; trues-MLEM for trues-to-activity reconstruction; and trues-MLTR for trues-to-attenuation reconstruction. Combinations of *n* individual algorithms form *n-algorithms*.

**Fig. 3:**
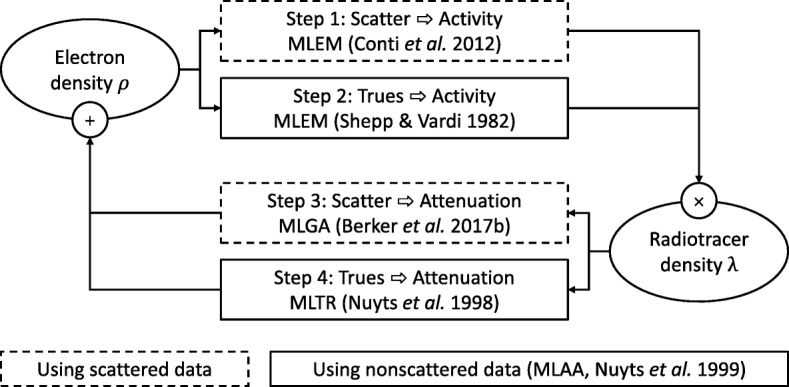
One iteration of joint estimation: an electron density estimate is used for attenuation correction in MLEM scatter-to-activity (step 1) and subsequent trues-to-activity (step 2) updates. Then, the activity distribution estimate is used as a source term in MLGA scatter-to-attenuation (step 3) and MLTR trues-to-attenuation (step 4) updates. In low-attenuation cases, only steps 2 and 3 are used in each iteration, while all steps are used in high-attenuation cases

As for S2A reconstruction, we distinguish two main cases: low and high attenuation. The general data flow per iteration is similar for both cases and is visualized in Fig. 3; radiotracer activity distribution *λ* and electron density *ρ* are repeatedly updated using the current estimate of the respective other quantity. For both cases, we re-optimized the MLGA step sizes to achieve stability.

#### 2-algorithms for low attenuation

**Fig. 4:**
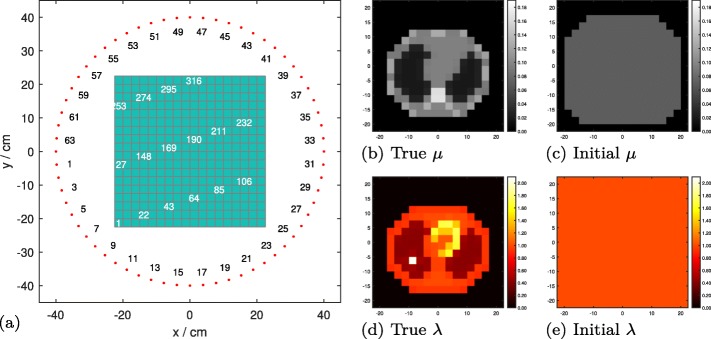
18×18-voxel simulation setup: **a** indices of detectors and voxels at their respective locations; **b** true and **c** initial *μ*-maps, respectively, in 1/cm; **d** true and **e** initial activity distributions, respectively, in arbitrary units; the initial activity distribution is used only for joint estimation (see the “*n*-algorithms for joint estimation” section). Human-sized phantom, axes scaled in cm

The idea of this subsection has been presented before [18, 29]. For low attenuation situations (e.g., with a spatial scale factor of 0.2 in Fig. 4), we interleave trues-MLEM with scatter-MLGA. The data flow in this part is similar to that proposed earlier [18]. We start with initial guesses for *ρ* and *λ*. In each iteration, plugging the current electron-density estimate $\vec {\rho }$ into $\boldsymbol {\tilde {U}}_{\rho }$, we use trues-MLEM to update the current activity estimate $\vec {\lambda }$ using the nonscattered data $\vec {z}$; then, we use the updated activity estimate to compute scatter-MLGA updates of $\vec {\rho }$.

In this part of the study, we aim to minimize the number of computationally expensive updates of the system matrix $\boldsymbol {\tilde {A}_{\lambda }}$. Therefore, we run 10 iterations of attenuation-corrected (using the current $\vec {\rho }$) trues-OSEM (with 4 data subsets) at a time, followed by 10 iterations of scatter-MLGA with 4 data subsets (the use of subsets in scatter-MLGA being studied in detail elsewhere [24]). This low-attenuation 2-algorithm is summarized as $(\text {trues-OSEM}^{10}_{4}) + (\text {scatter-MLGA}^{10}_{4})$, with a total of 20 sub-iterations per iteration. Since MLEM-OSL can replace scatter-MLGA for low attenuation, we also run $(\text {trues-OSEM}^{10}_{4}) + (\text {MLEM-OSL}^{10})$ on the same data for comparison.

#### 4-algorithms for low or high attenuation

For high attenuation (e.g., Fig. 4 at the original, that is, human spatial scale), we find it necessary to further consider activity information contained in scattered coincidences, as well as attenuation information contained in true coincidences. The former is achieved by the scatter-MLEM algorithm, the latter with the trues-MLTR algorithm with a relaxation factor of *η*=0.03. Iterations of different algorithms updating the same quantity are considered as one sub-iteration; all updates are applied subsequently (e.g., the trues-MLEM update used the estimate of *λ* as updated by the previous scatter-MLEM update; see Algorithm 1).

Not using any subsets, the high-attenuation 4-algorithm is noted (scatter-MLEM+trues-MLEM)+(scatter-MLGA+trues-MLTR), with two sub-iterations per iteration.

### Evaluation strategy

#### Evaluation data

We simulate data based on an 18×18-voxel version (*high resolution*, Fig. 4) of the human-sized chest cross-section phantom used previously [24], as well as the original one (9×9 voxels, *low resolution*, Fig. 14a). For the former, the voxel size is 25×25 mm^2^ and the radius of the 2-D PET scanner used to simulate a PET acquisition is 40 cm. For a rat-sized field of view (FOV), the phantom (and the scanner geometry) are uniformly scaled down by a factor of 0.2, all size relations remaining identical (5×5 mm^2^ pixel size, 8-cm detector radius)^6^. An intermediate, rabbit-sized FOV is obtained using a linear downscaling factor of 0.35. At all scales, the scanner is equipped with *N*_*d*_=64 equidistant detectors having *N*_*E*_=10 energy bins, of which 7 are effectively used: (511 keV, 460 keV] down to (204 keV, 153 keV].


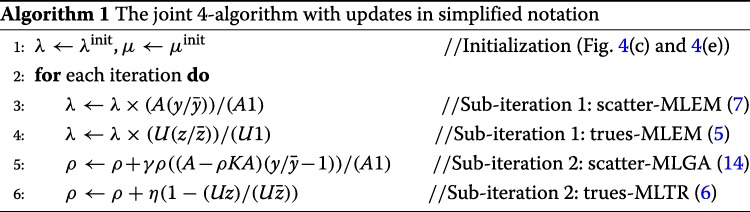


Single-scattered data is simulated by evaluating (2). Nonscattered data is simulated by (4) using a system matrix ***U***, each column *j* of which is constructed from the result of the MATLAB radon function [37] for a unity point source in *j*.

For all algorithms, the initial guess of *ρ* generously bounds the true object and is filled with the equivalent of *μ*=0.07/cm (Fig. 4c): this value ensures approximately correct attenuation correction factors for the first iteration of trues-to-activity reconstruction. For joint estimation, the initial activity is homogeneous throughout the FOV (Fig. 4e).

#### S2A reconstruction

The first part of this comparison of MLGA and MLEM-OSL is along the lines of earlier work comparing MLGA with 2BP [24], using additional simulation data with higher numbers of voxels than before. Therefore, both algorithms are applied to the (low and high resolution) data described above. Due to the small number of voxels, specific features of reconstructed images are of less interest; for the agreement between reconstructed images $\vec {x}$ with their respective references $\vec {x}^{\,\text {true}}$, we therefore report normalized mean squared errors (NMSE):

19 $$  \mathcal{S}(\vec{x}, \vec{x}^{\,\text{true}}) = \sum_{{i}} \left(x^{{i}} - x^{\,\text{true},{i}}\right)^{2} \Big/ \sum_{{i}} \left(x^{\,\text{true},{i}}\right)^{2}.  $$

##### FOV size variations

Both algorithms are applied to the data simulated at all three spatial scales (human: scale 1; rabbit: scale 0.35; rat: scale 0.2).

##### Reduced data

For MLEM-OSL, data is reduced by separating SORs into useful and less useful ones based on the aforementioned criterion, useful ones fulfilling:

20 $$  \max_{{j}} \sum_{s} \rho^{s} k^{{i}}_{s,{j}} \leq {1}.  $$

This criterion is evaluated using the current estimate of $\vec {\rho }$ in every iteration. SORs *i* which are to be left out are removed both from the data $\vec {y}$ (removing single data points) and the system matrix ***A***_*λ*_ (removing whole rows), and all computations are carried out with these reduced variables when working with reduced data.

##### Computational complexity and sparsity

Computational complexity of both algorithms is assessed by measuring run times on a consumer-grade laptop (Intel Core 2 Duo 2.8 GHz processor, 4 GB memory). Therefore, the simulation parameters are varied in two ways. First, with the number of voxels fixed at low resolution, we vary the number of detectors following *N*_*d*_=2^*n*^ with *n*∈{1,…,7}. Second, with the number of detectors fixed (at *N*_*d*_=32), we vary the number of voxels following 2^*n*^×2^*n*^ with *n*∈{1,…,5}; in terms of vector lengths, that corresponds to *N*_*e*_=*N*_*s*_=*N*_*t*_=4^*n*^. When varying the number of voxels, the voxel dimensions are adapted to maintain a constant spatial extent of the phantom.

For this part of the study, we choose constant activity and attenuation distributions (*λ*_*j*_=1,*μ*_*j*_=0.1/cm), with an initial *μ*_*j*_=0.05/cm. Since this choice implies a maximum population of the system matrix ***A***_*λ*_, we also determine what we term the *geometrical* density (fraction of non-null entries with flat activity) of ***A***_*λ*_ and $\underline {\boldsymbol {K}}$, respectively, which represent upper bounds for cases with less extended activity distributions.

#### Joint estimation

In joint estimation, in addition to computing NMSEs, we are interested in the evolution of several likelihood values. Attenuation- and activity-reconstruction algorithms are designed to maximize likelihoods given the true value of all other quantities. For scattered data, these are:

21a $$\begin{array}{*{20}l} \mathcal{L}^{\text{att}}_{\text{scatt}}\left({\rho^{\,\text{est}}}\right) &= \mathcal{L}_{y}\left(\vec{\bar{y}}\left(\lambda^{\,\text{true}}, \rho^{\,\text{est}}\right)\right) \end{array} $$

21b $$\begin{array}{*{20}l} \mathcal{L}^{\text{act}}_{\text{scatt}}\left({\lambda^{\,\text{est}}}\right) &= \mathcal{L}_{y}\left(\vec{\bar{y}}\left(\lambda^{\,\text{est}}, \rho^{\,\text{true}}\right)\right). \end{array} $$

However, in a joint-estimation setting, *λ*^true^ and *ρ*^true^ are generally not available. For the scattered and the true data, respectively, we therefore also track the *apparent* likelihoods, which are the quantities as seen by the optimization algorithms:

22a $$\begin{array}{*{20}l} \mathcal{L}^{\text{app}}_{\text{scatt}} &= \mathcal{L}_{y}\left(\vec{\bar{y}}\left(\lambda^{\,\text{est}}, \rho^{\,\text{est}}\right)\right) \end{array} $$

22b $$\begin{array}{*{20}l} \mathcal{L}^{\text{app}}_{\text{trues}} &= \mathcal{L}_{z}\left(\vec{\bar{z}}\left(\lambda^{\,\text{est}}, \rho^{\,\text{est}}\right)\right). \end{array} $$

We then study the following combinations of data and algorithms.

##### 2-algorithms for low attenuation

We apply $\left (\text {trues-OSEM}^{10}_{4}\right) + \left (\text {scatter-MLGA}^{10}_{4}\right)$ to the high-resolution, low-attenuation data. To verify that MLEM-OSL can replace MLGA, we also apply $\left (\text {trues-OSEM}^{10}_{4}\right) + \left (\text {MLEM-OSL}^{10}\right)$ to the same data.

##### 4-algorithms for low and high attenuation

We first compare the 4-algorithm, (scatter-MLEM+trues-MLEM)+(scatter-MLGA+trues-MLTR), to the 2-algorithm in terms of performance on the high-resolution, low-attenuation data; then, we apply only the 4-algorithm to high-resolution, high-attenuation data.

##### 4-algorithm to resolve MLAA crosstalk

During initial studies with a low-resolution object at the human scale (Fig. 14a), traditional MLAA ($\text {trues-MLEM}^{1}_{1} + \text {trues-MLTR}^{1}_{1}$) converged to an apparent local maximum of the likelihood (Fig. 14c). Therefore, we use the values of *ρ* and *λ* at this point to initialize the 4-algorithm.

#### Implementation

All algorithms are implemented in MATLAB (R2019a; The MathWorks, Natick, MA, USA). The Appendix describes the use of sparse matrices in evaluating likelihoods and gradients. In trues- and scatter-to-attenuation algorithms, instead of adding nonnegativity constraints, we set *ρ*^*j*^← max{0,*ρ*^*j*^} after each update.

## Results

### S2A reconstruction

**Fig. 5:**
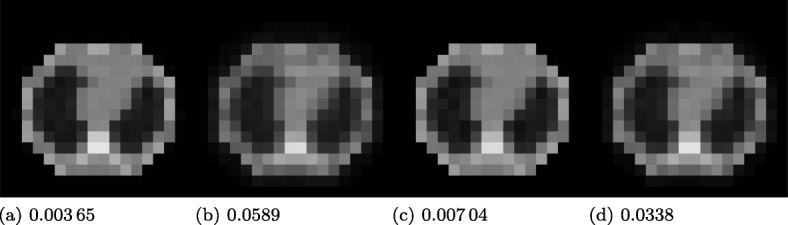
Estimated 18×18-voxel *ρ*-maps at rat scale (see Fig. 6c, right) and their NMSEs after 50 iterations of four S2A reconstruction algorithms, respectively: **a** MLGA, MLEM-like step size, **b** MLEM-OSL, **c** MLGA, constant step size., **d** MLGA, scaled step size

In this section, we verify the theoretical findings using NMSEs of reconstructed *ρ*-maps, of which we present some examples in Fig. 5.

**Fig. 6:**
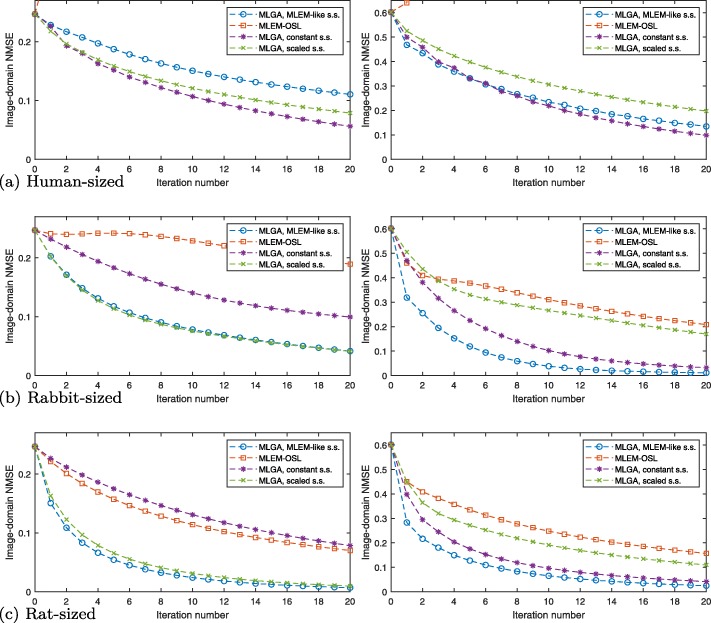
Comparison of algorithms, with the same phantom at **a** human size, **b** rabbit size, **c** rat size. NMSE of *ρ* in the image domain as a function of iteration number for different algorithms. Left, 9×9 voxels; right, 18×18 voxels. Note the quick divergence of MLEM-OSL towards infinity at human scale; see Fig. 7a for an extended vertical plot range

**Fig. 7:**
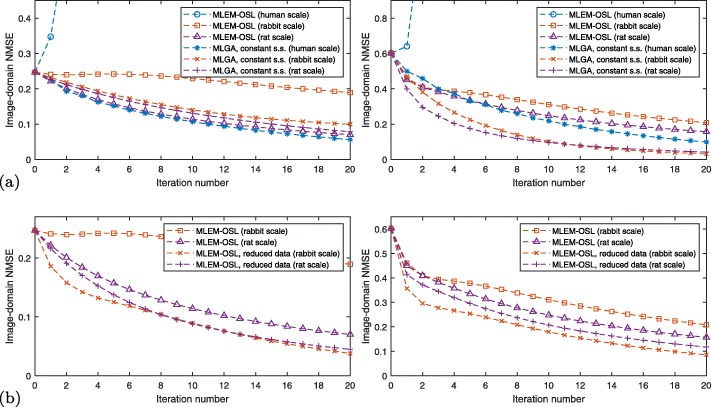
Influence of FOV scale and reduced data. NMSE of *ρ* in the image domain as a function of iteration number for **a** MLEM-OSL vs. MLGA with a constant step size in FOVs of various sizes and **b** MLEM-OSL with full vs. reduced data (rabbit-sized and rat-sized FOV). Left, 9×9 voxels; right, 18×18 voxels

Figure 6a shows the NMSEs of *ρ* for the human-sized phantom and system, for both low and higher resolution. All variants of MLGA converge to the correct solution as all NMSE curves tend to zero, while MLEM-OSL does not; MLGA with the MLEM-like step size is the fastest algorithm in both cases.

#### FOV size variations

Figure 6b and c show the data for the same phantom and system at rabbit and rat sizes, respectively. In these cases, all algorithms converge to the correct solution; generally, MLGA with the MLEM-like step size is among the fastest.

As summarized in Fig. 7a, MLEM-OSL converges well with a rat-sized FOV, less rapidly (and nonmonotonously) so with a rabbit-sized FOV, and not at all with a human-sized FOV despite otherwise identical simulations. This dependence of convergence, and convergence rates, on the spatial scale of the simulated phantom is less pronounced with MLGA which, even with a constant step size as an example, converges faster than MLEM-OSL in most cases.

#### Reduced data

Figure 7b verifies the hypothesis that MLEM-OSL scale dependence (and hence convergence) is improved by ignoring high-attenuation SORs in the data and the system matrix using (20). In fact, decreasing the dimensionality of the problem in this way leads to an increase in convergence speed for MLEM-OSL.

#### Computational complexity and sparsity

**Fig. 8:**
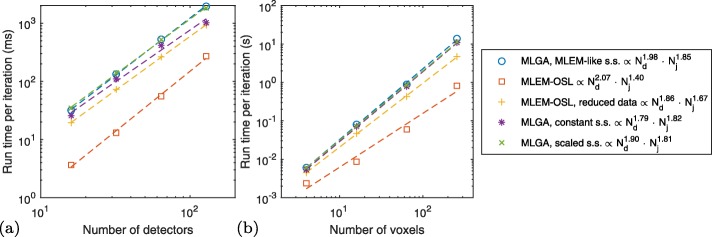
Computational complexity: run times per iteration of each algorithm using the fully populated phantom, with *N*_*E*_=4 and **a** *N*_*j*_=9×9, as a function of *N*_*d*_ from 16 to 128, **b** *N*_*d*_=32, as a function of *N*_*j*_ between 4×4 and 16×16

Figure 8 visualizes the run times per iteration of all algorithms in the fully populated geometry as a function of the number of detectors *N*_*d*_ and the number of voxels *N*_*j*_, respectively. The figure legends also include fitted power laws $\left (N_{d}^{a} N_{j}^{b}\right)$. While most algorithms show $\mathcal {O}\left (N_{d}^{2}\right)$ behavior, in terms of *N*_*j*_, the exponents range from 1.40 (MLEM-OSL) to 1.85 (MLGA, MLEM-like step size).

Tables 2 and 3 summarize the density of $\underline {\boldsymbol {K}}$ and ***A***_*λ*_ for the experiments shown in Fig. 8. Generally, the fraction of nonzero entries in both quantities decreases with increasing numbers of detectors or voxels.

**Table 2:** Geometrical density (in %) of $\underline {\boldsymbol {K}}$ and ***A***_*λ*_ with *N*_*j*_=9×9,*N*_*E*_=4

*N*_*d*_	2	4	8	16	32	64	128
$\underline {\boldsymbol {K}}$	4.30	4.30	3.80	3.16	2.78	2.15	1.42
***A***_*λ*_	33.6	34.5	26.8	22.1	18.9	14.3	9.19

Flat activity sources *λ* covering the complete FOV were used for ***A***_*λ*_ to establish an upper bound

**Table 3:** Geometrical density as in Table 2 for *N*_*d*_=32 and *N*_*E*_=4

*N*_*j*_	2×2	4×4	8×8	9×9	16×16
$\underline {\boldsymbol {K}}$	1.31	2.41	2.79	2.78	1.85
***A***_*λ*_	2.25	7.76	17.4	18.9	23.0

### Joint estimation

#### 2-algorithms for low attenuation

**Fig. 9:**
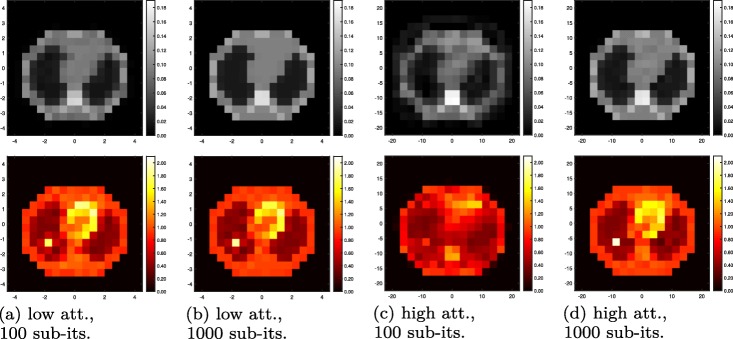
Joint estimation results for low-attenuation (rat-sized, left) and high-attenuation (human-sized, right) phantoms (as in Fig. 4). Reconstructed electron density maps (top) and activity images (bottom) after (**a**) 100 sub-iterations (5 iterations) of the 2-algorithm; (**b**) 1000 sub-iterations (50 iterations) of the 2-algorithm; (**c**) 100 sub-iterations (50 iterations) of the 4-algorithm; (**d**) 1000 sub-iterations (500 iterations) of the 4-algorithm

**Fig. 10:**
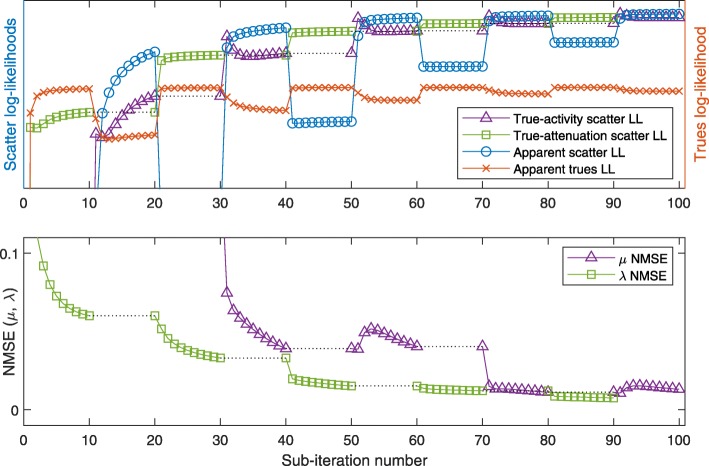
2-algorithm at low attenuation: log-likelihoods (LL) of (*μ*,*λ*) (with respect to the scattered and true data), and normalized mean square errors (NMSE) of *μ* and *λ*, respectively, during the first 100 sub-iterations (5 iterations) of the low-attenuation 2-algorithm. Note that the trues-MLEM activity updates [sub-iterations 0 to 10, 20 to 30, etc.] are supposed to increase the trues LL, explaining decreases in the scatter LLs, and vice versa

The resulting images of the 2-algorithm for the low-attenuation phantom are shown after 100 and 1000 sub-iterations, respectively, in Fig. 9a and b. For the evolution of true and apparent likelihoods, we refer to Fig. 10: this plot shows the NMSE of *μ* and *λ* as a function of sub-iterations, indicating the alternating updates of activity and attenuation, and similarly, the ideal log-likelihoods (LL) using the current estimate of one quantity and the true value of the respective other quantity, respectively. Finally, the apparent LLs of scattered and nonscattered data, based on both estimated activity and estimated attenuation, are plotted.

**Fig. 11:**
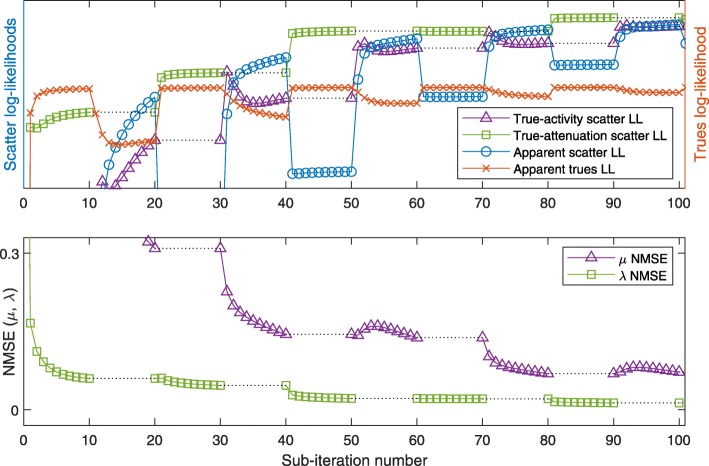
2-algorithm at low attenuation, as in Fig. 10, with MLEM-OSL replacing scatter-MLGA

In summary, the 2-algorithm converges towards the true activity and attenuation, even though true NMSE and apparent LL curves are nonmonotonous in parts. Figure 11 confirms that MLEM-OSL can replace scatter-MLGA in joint estimation, at low-attenuation and at the cost of reduced convergence speed.

#### 4-algorithm for low or high attenuation

**Fig. 12:**
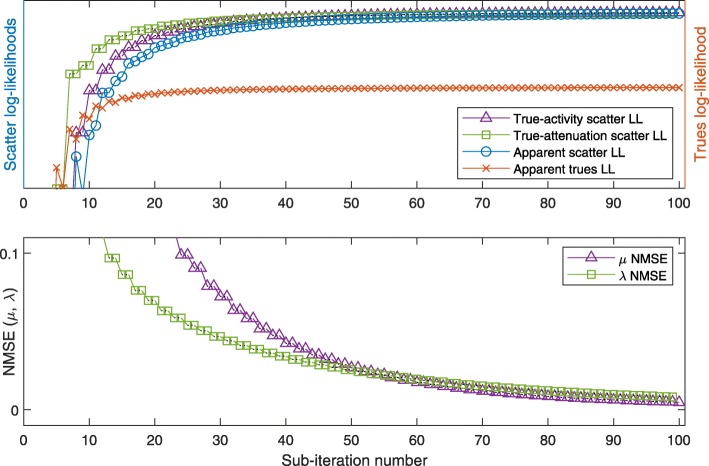
4-algorithm at low attenuation, as in Fig. 10, the 100 sub-iterations shown representing 50 iterations of the 4-algorithm

Figure 12 shows the results for the 4-algorithm in the low-attenuation case. Due to the fact that each iteration consists of only 2 sub-iterations (2 updates each), the LL and NMSE curves appear smoother than the same curves for the 2-algorithm.

**Fig. 13:**
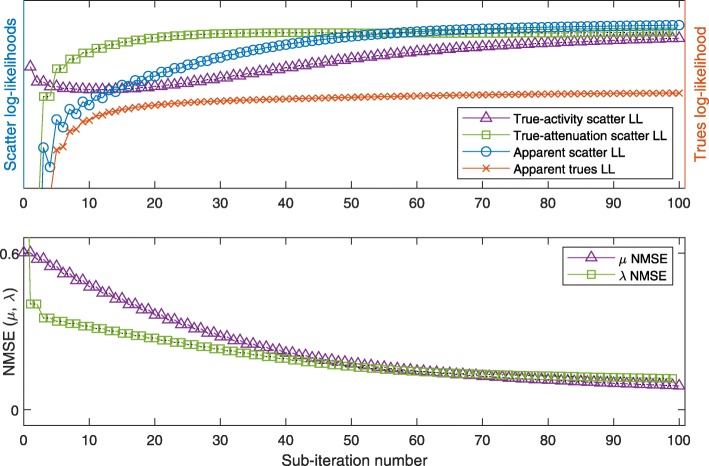
4-algorithm at high-attenuation as in Fig. 12

Applied to the same phantom at high attenuation, the 4-algorithm converges slower, but in a similarly smooth way as for low attenuation to the true activity and attenuation (Figs. 9c, d and 13): some nonmonotonicity remains, both at the sub-iteration level (for apparent LLs) and at the scale of dozens of sub-iterations (e.g., the true-activity LL early on or the true-attenuation LL, for which we note that it increases for later sub-iterations).

**Fig. 14:**
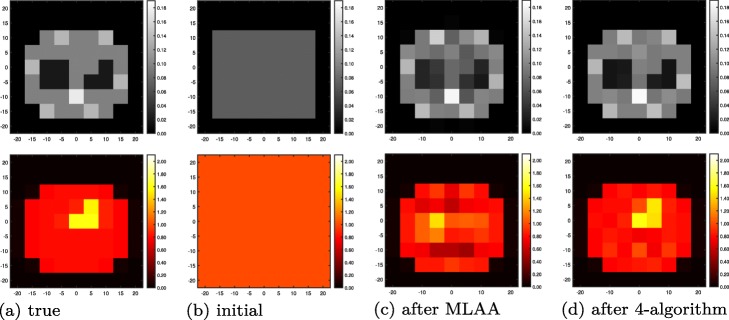
Crosstalk study of a low-resolution, high-attenuation (human-sized) phantom (as in Fig. 4): **a** true activity and attenuation; **b** activity and attenuation used to initialize MLAA; **c** activity and attenuation after apparent MLAA convergence, used to initialize the 4-algorithm; and **d** activity and attenuation after 1000 sub-iterations (500 iterations) of the 4-algorithm

#### 4-algorithm to resolve MLAA crosstalk

Finally, Fig. 14 shows the results of the crosstalk study. While the MLAA 2-algorithm is stuck in an apparent local maximum (Fig. 14c),^7^ the proposed 4-algorithm is able to not only avoid, but escape from this local maximum and converges towards the true solution (Fig. 14d).

## Discussion

We have studied reconstruction of attenuation information from scattered coincidences in PET. Unlike other problems regarding reconstruction of activity or attenuation from nonscattered or scattered coincidences, the problem at hand is unique in that the reconstructed quantity appears twice in the measurement equation (2). The problem is therefore nonlinear, with the degree of nonlinearity depending on attenuation, which in mostly water-like objects implies spatial scale^6^.

### S2A reconstruction

We have interpreted a recent take on the problem [18] as MLEM with a one-step-late update of the attenuated system matrix. MLEM-OSL, ignoring the dependence of $\boldsymbol {\tilde {A}}_{\lambda,\rho }$ on *ρ* in computing a new estimate, has been derived by linearizing the nonlinear measurement equation; however, the fact that MLEM-OSL thus relies on a linear relationship between electron density and scattered coincidences impacts performance with high attenuation (Fig. 7a). An important result is therefore the characterization of the spatial-scale problem, which is complementary to the intensity-scale problem described earlier for joint estimation of activity and attenuation from only nonscattered data [15, 39]. Another result is a potential nonuniqueness of the isolated S2A problem indicated in Fig. 16: fortunately, the same figure indicates that combined scattered and nonscattered data do not necessarily feature the same nonuniqueness. Also, we hypothesize that additional voxels and detectors help further resolve nonuniqueness.

We have studied a maximum-likelihood gradient-ascent method for attenuation-map reconstruction based on the full, nonlinear data log-likelihood. Since the step size ignores the dependence of $\boldsymbol {\tilde {A}}_{\lambda,\rho }$ on *ρ*, MLGA does not feature the provable monotonicity of the likelihood that MLEM offers in activity reconstruction from nonscattered coincidences. However, MLGA has advantages over MLEM-OSL, where ignoring said dependence leads to a wrong *direction* of the update and results in instabilities: the impact on MLGA is noticeably smaller.

Application to simulated data confirmed that in larger objects, MLGA outperforms MLEM-OSL. Nonetheless, MLEM-OSL is a simple and fast algorithm for rabbit- or rat-sized objects, while MLGA may require additional speedup [24].

By characterizing high attenuation, we have found a criterion to separate low- from high-attenuation data and improve MLEM-OSL convergence speed (Fig. 7b). Thus, one strategy to decrease the size of system matrices and tensors, and thus computational complexity, lies in choosing the most useful SORs from the full data. Here, we only briefly mention the possibilities of optimizing lower and upper energy photon thresholds in the detectors, redefining the detector’s transaxial acceptance angle, or selecting SORs intersecting specific parts of the subject [17, 24].

While MLGA converges, we find it to be more computationally complex than MLEM-OSL (Fig. 8). Also, MLEM-OSL with reduced data is more complex than using the full data: this could be remedied by stopping to re-evaluate (20) after some iterations.

### Joint estimation

MLGA, or MLEM-OSL, are not primarily meant as stand-alone algorithms, as they assume knowledge of the unknown *λ*, mandating a joint (*λ*,*ρ*) estimation scheme. This scheme is similar to traditional MLAA for estimation of (*λ*,*μ*) from nonscattered coincidences, where knowledge of *λ* is assumed by MLTR. Along the same lines, we have focused on using MLGA in joint *n*-algorithms. Just as MLAA iterates back and forth between MLEM and MLTR, reconstructing one quantity (*λ* or *μ*) while keeping the other (*μ* or *λ*, respectively) fixed, our proposed 2-algorithm iterates back and forth between trues-MLEM and scatter-MLGA. Another viable scheme encompassing all available data has been presented in the form of the scatter-MLEM/trues-MLEM/MLGA/MLTR 4-algorithm, using both true and scattered coincidences for estimation of both *λ* and *ρ*.

For low-attenuation data, the simple 2-algorithm may be sufficient: in this case, MLEM-OSL may serve as a drop-in replacement for MLGA, however with decreased convergence speed (compare Figs. 10 and 11). This result is compatible with the results for S2A reconstruction (Fig. 6b and c). The more sophisticated 4-algorithm enables joint reconstruction with high-attenuation as well as low-attenuation data. In addition, this 4-algorithm can employ the scatter information to escape from a nonoptimal fix point of MLAA (Fig. 14d).

The plots of apparent (using estimated activity and attenuation) and ideal likelihoods indicate several nonmonotonicities that are overcome by the combination of algorithms. In particular, in the 2-algorithm, increasing the apparent scatter likelihood with MLGA updates of the attenuation often decreases the true-activity scatter likelihood, limiting the number of repeated MLGA updates that can be concatenated. This observation is one reason for choosing only a single update of each algorithm in each iteration of the 4-algorithm (Algorithm 1).

### Limitations

This study has several limitations in the simplicity of the simulations: in particular, in neglecting detector scatter, multiple scatter, and energy-measurement uncertainties; in using the same forward model for the simulation as for the reconstruction; and in using low-dimensional objects and scanners.

In reality, the use of scattered photons is complicated by the fact that the detected signal of a nonscattered 511 keV photon, when it deposits only part of its energy in the detector, resembles that of a lower-energy, object-scattered photon [34]. One solution may be the use of an object-scatter energy window above the Compton edge (at 341 keV) and below the photopeak, which is virtually free of detector-scattered photons ([40], Fig. 1). This highest-possible energy window also has a lower contribution of multiply scattered photons [34].

Furthermore, photon energy measurements suffer from uncertainties in the range of 10% FWHM in state-of-the-art PET scanners. This uncertainty leads to blurred estimation of potential scattering locations in the object. So far, it is unclear exactly which energy resolution is required to successfully use this approach in practice, although some comparisons have been made for S2A reconstruction ([17], Fig. 11). In joint *n*-algorithms, separation of scattered and unscattered photons will be of importance in all sub-algorithms. New detector materials, such as LaBr_3_ [41] or cadmium zinc telluride [42], might be needed.

Another limitation is that specific findings may not be generalizable to arbitrary scanner geometries; for example, 3-D geometries may exhibit different, presumably much sparser system quantities ***A***_*λ*_ and $\underline {\boldsymbol {K}}$. We expect that this increasing sparsity partially offsets the (otherwise unmanageable) size increase of these quantities with growing number of voxels, detectors, and energy bins.

It should be noted that due to computational complexity, 2-D considerations are not uncommon in recent studies regarding image reconstruction from scattered photons [20, 26, 43]. Furthermore, a more sophisticated imaging model that offers more realistic system matrix components ***A***_*λ*_ and $\underline {\boldsymbol {K}}$ would be subject to the same measurement equations and lead to the same derivation of MLGA. So while specific convergence rates may vary with the density and condition number of those quantities, we expect the overall conclusions to prevail in more realistic settings. Finally, noise will have to be considered in future studies; currently, it is challenging to determine realistic noise levels for these nonrealistic types of objects and scanners.

### Outlook

In this paper, we have used MLGA as a S2A building block in the context of joint estimation. It might be possible to find improved algorithms: for example, one might pursue one of the many paths that lead to the MLEM update equation for an algorithm which features more of the well-known properties of MLEM. This may include the minorize/maximize (MM) algorithm [44], of which regular MLEM is one special case. Following earlier incomplete-data formulations [32], one might define complete data that involve not only the emission location, but also the scattering location of every coincidence; this may result in a formulation similar to that for joint estimation from nonscattered data [45]. Algorithms that use the formulation of the Hessian of the log-likelihood may also be of value without requiring inversion of the full Hessian during image reconstruction, as has been shown recently [25, 26].

TOF information might improve S2A reconstruction by increasing the sparsity of $\underline {\boldsymbol {K}}$ (as some scattering locations on the surface of the football may not be compatible with the emission locations indicated by a TOF measurement) and further improving the condition of ***A***_*λ*_ (by reducing the number of emission voxels over which to compute $\sum _{e} b^{{i}}_{s,e} \lambda ^{e}$). Our study does not simulate, or incorporate, TOF measurements, as the amount of additional information from TOF is nonetheless limited in attenuation reconstruction compared to activity reconstruction: even with perfect TOF information, the surface of potential scattering locations (and hence the density of $\underline {\boldsymbol {K}}$) will hardly be reduced by more than a few times, on average, as most broken LORs compatible with a non-TOF coincidence will also be compatible with the TOF coincidence. Therefore, the primary way for TOF information to find its way into this problem may be through activity-reconstruction building blocks (TOF-trues-MLEM and TOF-scatter-MLEM) and the estimate of the activity distribution they provide—similar to how TOF-MLAA benefits from TOF information without the MLTR algorithm using it explicitly.

Regarding the impact of the results outside of PET imaging, we have achieved a definition of data being more or less compatible with a linearization of the measurement equation that may be applied in external Compton scatter imaging, in which a number of ways have been tried to solve a structurally similar measurement equation [28]—compare in particular (2) to ([46], Eq. 3) or ([47], Eq. 1). Furthermore, full knowledge of the “source” distribution is given in CT and other transmission imaging modalities, where scattered radiation could be similarly exploited if discerned by energy measurements, such as in multi-energy (spectral) CT [48].

## Conclusion

In reconstruction of attenuation information from scattered PET coincidences, maximum-likelihood gradient-ascent algorithms provide faster convergence and convergence in more diverse setups than MLEM-OSL, for which we have presented both analytic and experimental evidence: MLGA converges across all spatial scales, while MLEM-OSL may only converge with smaller objects. Nonetheless, MLEM-OSL can be a lower-complexity alternative to MLGA. We have defined a numerical criterion to determine when the simpler and more efficient MLEM-OSL can be used and described how its performance can be improved by reducing data based on said criterion. Finally, joint estimation of activity and attenuation from scattered and nonscattered coincidences has been presented using either MLGA or MLEM-OSL, in particular, in an example where MLAA fails to converge to the correct solution.

## Appendix

## Derivation of discrete measurement equations

In the previous notation of the measurement equation ([17], Eq. 5) and its derivation ([49], Eq. 11), the continuous measurement equation is:

23 $$ \begin{aligned} \bar{N}^{S} (d_{1}, E_{1}, d_{2}) &\approx T \cdot \Delta E_{1} \cdot \left(\frac{b}{2} \right)^{3} \cdot \frac{(\cos \theta_{1} - 2)^{2}}{E_{0} \cdot \sin \theta_{1}} \cdot \left.\frac{\mathrm{d}^{2} \sigma_{\text{KN}}}{\mathrm{d}^{2} \Omega} \right|_{\theta_{1}} \cdot \int_{-\theta_{1}}^{+\theta_{1}} \!\!\!\! \mathrm{d} \omega_{S} \int_{0}^{2 \pi} \!\! \mathrm{d} \varphi_{S}\\ &\left[\frac{(\cos \omega_{S} - \cos \theta_{1})^{2}}{\sin^{4} \theta_{1}} \cdot \rho_{e}(\vec{x}_{S}) \cdot \int_{L(\vec{x}_{S}, \vec{x}_{2})} \!\! \!\!\!\! \!\!\!\! \!\!\!\! \lambda (\vec{x}_{\lambda}) \mathrm{d} r_{\lambda}^{S} \cdot \Omega_{1} (\vec{x}_{S}) \cdot \Omega_{2} (\vec{x}_{S})\right.\\ &\cdot \exp \left(- \int_{L (\vec{x}_{2},\vec{x}_{S})} \!\!\!\! \!\!\!\! \!\!\!\! \!\!\! \mu (\rho_{e} (\vec{x})) \mathrm{d} x \,\, - \int_{L (\vec{x}_{S},\vec{x}_{1})} \!\!\!\! \!\!\!\! \!\!\!\! \!\!\! \mu_{E_{1}} (\rho_{e} (\vec{x})) \mathrm{d} x\right)\\ &\left.{\vphantom{\frac{(\cos \omega_{S} - \cos \theta_{1})^{2}}{\sin^{4} \theta_{1}}}}\cdot \mathcal{E} (d_{1}, E_{1}, {\vec{x}_{1} - \vec{x}_{S}}) \cdot \mathcal{E} (d_{2}, E_{0}, {\vec{x}_{2} - \vec{x}_{S}}) \right]. \end{aligned}  $$

We refer to the original publications for more context of this equation: $\bar {N}^{S} (d_{1}, E_{1}, d_{2})$, number of scattered coincidences detected in detectors *d*_1_ (with energy *E*_1_) and *d*_2_; *T*, acquisition time; *ΔE*_1_, energy bin width; *b*, length of baseline connecting *d*_1_ and *d*_2_;*θ*_1_, scattering angle associated with *E*_1_;*E*_0_, 511 keV; *σ*_KN_, Klein-Nishina scattering cross-section; *ω*_*S*_,*φ*_*S*_, angles parameterizing the surface of scattering locations $\vec {x}_{s}$; *ρ*_*e*_, electron density; *λ*, radiotracer density; *L*, a line segment connecting two points; *Ω*_1_, *Ω*_2_, detector solid angles seen from $\vec {x}_{S}; \mu, \mu _{E_{1}}$, linear attenuation coefficient at *E*_0_ and *E*_1_; $\mathcal {E}$, photon detection sensitivity as a function of detector element, photon energy, and angle of incidence. While most terms have direct physical interpretations:

24 $$ \left(\frac{b}{2} \right)^{3} \cdot \frac{(\cos \omega_{S} - \cos \theta_{1})^{2}}{\sin^{4} \theta_{1}} \quad\text{and}\quad \frac{(\cos \theta_{1} - 2)^{2}}{E_{0} \cdot \sin \theta_{1}}  $$

arise from the Jabobian determinant of the coordinate transformation from Cartesian coordinates to surface parameter angles and account for a change of variables from polar scatter angle to photon energy, respectively [49].

In discretizing and changing notation, we apply the following mapping:

25 $$\begin{array}{*{20}l}  {i} = (d_{s}, d_{n}, E) ~\longleftarrow~& (d_{1}, E_{1}, d_{2}) \end{array} $$

26 $$\begin{array}{*{20}l}  \bar{y} ~\longleftarrow~& \bar{N}^{S} \end{array} $$

27 $$\begin{array}{*{20}l}  c^{{i}} ~\longleftarrow~& T \cdot \Delta E_{1} \cdot \left(\frac{b}{2} \right)^{3} \cdot \frac{(\cos \theta_{1} - 2)^{2}}{E_{0} \cdot \sin \theta_{1}} \cdot \left. \frac{\mathrm{d}^{2} \sigma_{\text{KN}}}{\mathrm{d}^{2} \Omega} \right|_{\theta_{1}} \end{array} $$

28 $$\begin{array}{*{20}l}  \sum_{s} \hat{c}^{{i}}_{s} ~\longleftarrow~& \int_{-\theta_{1}}^{+\theta_{1}} \!\!\!\! \mathrm{d} \omega_{S} \int_{0}^{2 \pi} \!\! \mathrm{d} \varphi_{S} \end{array} $$

$$\begin{array}{*{20}l}  \hat{c}^{{i}}_{s} ~\longleftarrow~& \frac{(\cos \omega_{S} - \cos \theta_{1})^{2}}{\sin^{4} \theta_{1}} \cdot \Omega_{1} (\vec{x}_{S}) \cdot \Omega_{2} (\vec{x}_{S}) \notag \end{array} $$

29 $$\begin{array}{*{20}l} & \cdot \mathcal{E} (d_{1}, E_{1}, {\vec{x}_{1} - \vec{x}_{S}}) \cdot \mathcal{E} (d_{2}, E_{0}, {\vec{x}_{2} - \vec{x}_{S}}) \end{array} $$

30 $$\begin{array}{*{20}l}  \rho^{s} ~\longleftarrow~& \rho_{e} (\vec{x}_{S}) \end{array} $$

31 $$\begin{array}{*{20}l}  \sum_{e} \tilde{c}^{{i}}_{s,e} ~\longleftarrow~& \int_{L (\vec{x}_{S}, \vec{x}_{2})} \!\! \!\!\!\! \!\!\!\! \!\!\!\! \mathrm{d} r_{\lambda}^{S} \end{array} $$

32 $$\begin{array}{*{20}l}  \lambda^{e} ~\longleftarrow~& \lambda (\vec{x}_{\lambda}) \end{array} $$

33 $$\begin{array}{*{20}l}  \sum_{t} l^{{i}}_{s,t} ~\longleftarrow~& \int_{L (\vec{x}_{2},\vec{x}_{S})} \!\!\!\! \!\!\!\! \!\!\!\! \!\!\! \mathrm{d} x \,\, + \int_{L\left(\vec{x}_{S},\vec{x}_{1}\right)} \!\!\!\! \!\!\!\! \!\!\!\! \!\!\! \left(\mu_{E_{1}}(\rho_{e}(\vec{x})) \big/ \mu(\rho_{e}(\vec{x}))\right) \mathrm{d} x \end{array} $$

34 $$\begin{array}{*{20}l}  \mu^{t} ~\longleftarrow~& \mu (\rho_{e} (\vec{x})) \end{array} $$

**Fig. 15:**
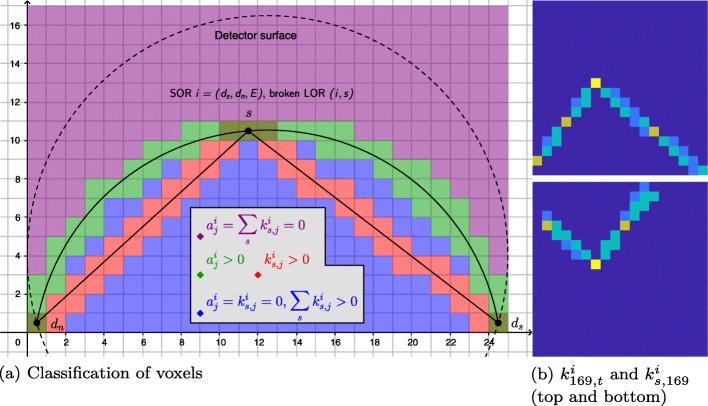
**a** Classification of image-space voxels *j* contributing to a measurement on SOR *i*; in terms of $\smash {a^{{i}}_{{j}}}$ (contribution through scattering), $\smash {\sum _{s} k^{{i}}_{s,{j}}}$ (contribution through attenuation) and $\smash {k^{{i}}_{s,{j}}}$ (contribution through attenuation specifically along broken LOR (*i*,*s*)). **b** Comparison of different slices of $\underline {\boldsymbol {K}}$: $\smash {k^{i}_{169,t}}$ as a function of *t* (top) and $\smash {k^{{i}}_{s,169} }$ as a function of *s* (bottom) for *i*=(*d*_*s*_,*d*_*n*_,*E*)=(24,8,5). Top: with the endpoints of the SOR *i* at the bottom left (detector 8) and bottom right (detector 24), and a scattering location in a central voxel (index 169, see Fig. 4), $\smash { k^{{i}}_{169,t} }$ represents the attenuation weights of voxels *t* along the (one) broken LOR (*i*,*s*=169). Bottom: by contrast, $\smash {k^{{i}}_{s,169}}$ shows the attenuation weights of the (one) voxel *t*=169 along various, different broken LORs (*i*,*s*) with the same endpoints, but different scattering locations *s*

Two remarks are in order. First, $\hat {c}^{{i}}_{s}$ serves a double purpose of both defining the domain of summation over potential scattering locations $\vec {x}_{s}$ (28) and including weighting factors (29). Due to the former, it can be stored efficiently as a sparse matrix; this holds true for $c^{{i}}_{s,e}$ and $l^{{i}}_{s,t}$ as well. Second, in the mapping (33), $l^{{i}}_{s,t}$ represents an *effective* attenuation length by encoding the differences in attenuation coefficients seen by scattered photons of different energies (*μ*_*E*_ at lower energy *E*) compared to nonscattered photons (*μ*=*μ*_511keV_). In other words, a voxel can have different lengths along different broken LORs. This eliminates the need to store different attenuation coefficients for the same voxel, assuming that *μ*_*E*_/*μ* is known. If one has *μ*∝*ρ*, that is, *μ*_*E*_(*ρ*)=*α*(*E*)·*ρ*, one finds

35 $$  \frac{\mu_{E}}{\mu} = \frac{\alpha(E)}{\alpha({511}\ \text{keV})},  $$

which can be determined knowing only *E*. Since *i* comprises the scattered photon energy *E* (25), and each broken LOR (*i*,*s*) defines which voxels *t* are part of the respective lower-energy section (Fig. 15a), the effective attenuation lengths $l^{{i}}_{s,t}$ can be computed. While this reasoning confirms that the shape of (2) does not impede taking into account energy-dependent attenuation, we did not do so here.

### Compression and simplification

In the above way, fully accounting for all terms and operators in (23), we obtain:

36 $$  \bar{y}^{{i}} = c^{{i}} \sum_{s} \hat{c}^{{i}}_{s} \cdot \rho^{s} \cdot \left(\sum_{e} \tilde{c}^{{i}}_{s,e} \cdot \lambda^{e}\right) \cdot \exp \left(- \sum_{t} l^{{i}}_{s,t} \cdot \mu^{t}\right).  $$

While in terms of computational complexity, it may be beneficial to compute $c^{{i}}, \hat {c}^{{i}}_{s}$, and $\tilde {c}^{{i}}_{s,e}$ separately; with regard to storage and algorithmic simplicity, one may prefer combining all these terms into $b^{{i}}_{s,e} = c^{{i}} \cdot \hat {c}^{{i}}_{s} \cdot \tilde {c}^{{i}}_{s,e}$. Continuing to assume that *μ*∝*ρ*, we set

37 $$  k^{{i}}_{s,t} := l^{{i}}_{s,t} \cdot \mu^{t} / \rho^{t} = l^{{i}}_{s,t} \cdot \alpha({511}\ \text{keV})  $$

to find

38 $$  \bar{y}^{{i}} = \sum_{s} \rho^{s} \cdot \left(\sum_{e} b^{{i}}_{s,e} \cdot \lambda^{e}\right) \cdot \exp \left(- \sum_{t} k^{{i}}_{s,t} \cdot \rho^{t}\right).  $$

This equation forms the basis of both activity and attenuation reconstruction.

#### Activity reconstruction

For the simpler case of activity reconstruction [34], we consider $\vec {\rho }$ as a parameter for attenuation correction. Then, changing the order of summation in (38):

39 $$  \bar{y}^{{i}} = \sum_{e} \lambda^{e} \cdot \left(\sum_{s} \rho^{s} \cdot b^{{i}}_{s,e} \cdot \exp \left(- \sum_{t} k^{{i}}_{s,t} \cdot \rho^{t}\right) \right),  $$

we recognize that (39) can be written as a matrix-vector product:

40 $$  \vec{\bar{y}} = \boldsymbol{\tilde{A}}_{\rho} \vec{\lambda},  $$

where $\boldsymbol {\tilde {A}}_{\rho }$ is the matrix with entries $\tilde {a}^{{i}}_{e} := \sum _{s} \rho ^{s} \cdot b^{{i}}_{s,e} \cdot \exp \left (- \sum _{t} k^{{i}}_{s,t} \cdot \rho ^{t}\right)$. Knowing (an estimate of) *ρ*, the linear nature of (40) allows application of the regular MLEM algorithm [32] to reconstruct or update *λ*, as has been described by [34].

#### Attenuation reconstruction

Focusing on attenuation reconstruction, we consider $\vec {\lambda }$ as a parameter instead. Then, $b^{{i}}_{s,e}$ and *λ*^*e*^ can be combined into ***A***_*λ*_ with entries $a^{{i}}_{s} := \sum _{e} b^{{i}}_{s,e} \cdot \lambda ^{e}$, yielding

41 $$  \bar{y}^{{i}} = \sum_{s} \rho^{s} \cdot a^{{i}}_{s} \cdot \exp \left(- \sum_{t} k^{{i}}_{s,t} \cdot \rho^{t}\right).  $$

By using element-wise operations (⊙ and $\overset {\circ }{\text {exp}}$) and by defining 3rd-order tensors $\underline {\boldsymbol {B}}$ and $\underline {\boldsymbol {K}}$ and the matrix $\boldsymbol {A}_{\lambda } := \underline {\boldsymbol {B}} \vec {\lambda }$, (38) and (41) can also be written as:

42 $$ \vec{\bar{y}} = \left(\left(\underline{\boldsymbol{B}} \vec{\lambda}\right) \odot \overset{\circ}{\text{exp}} \left(- \underline{\boldsymbol{K}} \vec{\rho}\right)\right) \vec{\rho} = \left(\boldsymbol{A}_{\lambda} \odot \overset{\circ}{\text{exp}} \left(- \underline{\boldsymbol{K}} \vec{\rho}\right)\right) \vec{\rho},  $$

respectively, where the latter is (2) as was to show.

### Unscattered coincidences

For (mostly) unscattered data, where the one value of *E* represents the photopeak energy window, an SOR reduces to an LOR, and $\tilde {a}^{i}_{e}$ is replaced by $\tilde {u}^{l}_{j}$ for activity reconstruction; similarly, in attenuation reconstruction, $a^{{i}}_{s}$ is replaced by $u^{l}_{j}$.

## Implementation using sparse matrices

$\underline {\boldsymbol {K}}$ and ***A***_*λ*_ are highly sparse, and so is $\underline {\boldsymbol {K}} \vec {\rho }$. However, since exp0=1, $\exp (- \underline {\boldsymbol {K}} \vec {\rho })$ is not sparse, which impedes storage of intermediate results as sparse matrices. Hence, we rewrite:

43 $$  \boldsymbol{A}_{\lambda} \odot \overset{\circ}{\text{exp}} \left(- \underline{\boldsymbol{K}} \vec{\rho}\right) = \boldsymbol{A}_{\lambda} \odot \left(\overset{\circ}{\text{exp}} \left(- \underline{\boldsymbol{K}} \vec{\rho}\right) - \vec{1}_{[{i}]} \otimes \vec{1}_{[s]}\right) + \boldsymbol{A}_{\lambda}  $$

and use the expm1 function [50] to compute the sparse $\boldsymbol {S} := \overset {\circ }{\text {exp}} (- \underline {\boldsymbol {K}} \vec {\rho }) - \vec {1}_{[{i}]} \otimes \vec {1}_{[s]}$, followed by ***A***_*λ*_⊙***S***+***A***_*λ*_. This maintains the sparsity in all intermediate results along the memory-efficient evaluation of (42).

## Multi-voxel interpretations of high attenuation

Motivated by the 1-D results around (17), we are interested in the sign of the gradient of the expected data. In component-wise notation, (12) reads:

44 $$  \partial \bar{y}^{{i}} / \partial \rho^{j} = \sum_{s} \left(\delta_{sj} - \rho^{s} k^{i}_{s,{j}}\right) a^{{i}}_{s} \exp \left(- \sum_{t} k^{i}_{s,t} \rho^{t}\right),  $$

with *δ*_*sj*_ the Kronecker delta. Based on the sign of this expression, we can identify distinct regions of the football-shaped SOR in Fig. 1. Figure 15a shows half a cross-sectional plane through the football,^8^ determined by *d*_*s*_, *s*, and *d*_*n*_. On the inside and outside of the football, the sign is determined mainly by the quantities $a^{{i}}_{{j}}$ and $k^{{i}}_{s, {j}}$, which represent the scattering contributions of a voxel *j* along an SOR *i* and the attenuating contributions of a voxel *j* along broken LORs (*i*,*s*), respectively.

**Outside** When both $a^{{i}}_{{j}} = 0$ and $\sum _{s} k^{{i}}_{s,{j}} = 0$ ($\Leftrightarrow k^{{i}}_{s,{j}} = 0 \ \forall s$), we find $\partial \bar {y}^{{i}} / \partial \rho ^{{j}} = 0$. The voxel *j* does not contribute to a measurement along *i*, neither through scattering nor through attenuation; in Fig. 15a, this region of voxels *j* corresponds to the violet area outside the football. Changing *ρ*^*j*^ does not influence $\bar {y}^{{i}}$.

**Fig. 16:**
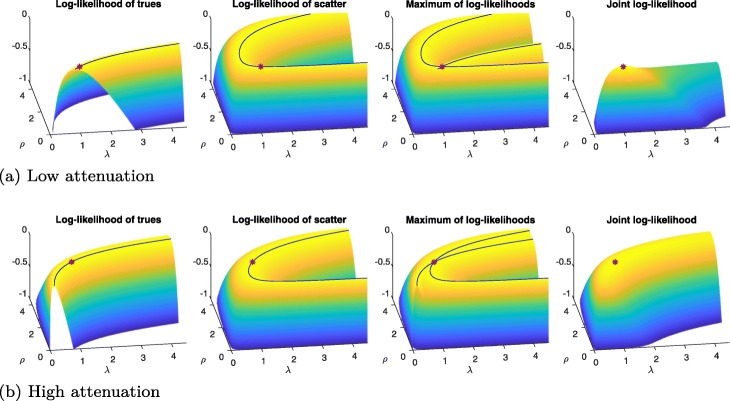
Log-likelihoods (individually normalized to [− 1,0]) in a single-voxel example for true and scattered coincidences, their maximum, and the joint likelihood of trues and scatter. **a** Low attenuation, *ρ*^true^=0.2. **b** High attenuation, *ρ*^true^=2. Red asterisks mark true maxima, respectively, while blue contour lines trace other maxima

**Inside** The football’s strict inside contributes to a measurement along SOR *i* only through attenuation. We have $a^{{i}}_{{j}} = 0$ (voxel *j* not contributing scatter to this SOR), but $\smash {\sum _{s} k^{{i}}_{s,{j}} > 0 }$ (voxel *j* attenuating on at least one broken LOR of this SOR). These are red and blue areas in Fig. 15a: red highlights where $\smash {k^{{i}}_{s,{j}} > 0 }$ for one particular *s*; blue areas where $\smash {k^{{i}}_{s,{j}} = 0 }$, but $\smash {k^{{i}}_{s',{j}} > 0 }$ for at least one other *s*^′^≠*s*. The result is $\partial \bar {y}^{{i}} / \partial \rho ^{{j}} < 0$: increasing *ρ* in *jdecreases* the expected number of coincidences on *i*.

Thus, when taking these attenuating effects into account, e.g., in an iterative update, it would be more appropriate to speak of a *volume* rather than a surface of response; we still hold on to the term SOR here.

**Surface** Defined by $\smash {a^{{i}}_{{j}} > 0 }$ and marked green in Fig. 15a, the football’s hull is the most interesting segment. These voxels are the only ones with scattering contributions, with potentially additional attenuating contributions (overlap between green and red regions). If we had $\smash {\sum _{s} \rho ^{s} k^{i}_{s,{j}} = 0 }$, then $\partial \bar {y}^{i} / \partial \rho ^{j} \geq 0$ since *j* would only contribute through scattering. However, with $\smash {a^{i}_{j} > 0 }$, we certainly have $\smash {k^{i}_{{j},{j}} > 0 }$: a scattering voxel is always on the broken LOR through it, and hence attenuating. Thus, if *ρ*^*j*^>0 (which means *j* lies within the object), the sign of $\partial \bar {y}^{{i}} / \partial \rho ^{{j}}$ is more complex to determine. Besides that, we have only little general information about $k^{{i}}_{s,{j}}$ for *s*≠*j*; this greatly depends on the object (low vs. high attenuation), the geometry of *i* and (*i*,*s*), as well as the discretization strategy.

We therefore aim for a definition of “high attenuation.” For now, we will therefore ignore differences between voxels that are both inside the object (*ρ*^*j*^>0) and on the surface of SOR *i* ($a^{{i}}_{{j}} > 0$), and refer to them by their number *n* and averages $\bar {a}, \bar {k}$, and $\bar {\rho }$. This way, we simplify (44) to:

45 $$ \partial \bar{y}^{{i}} / \partial \rho^{{j}} \approx \bar{a} \exp(- n \bar{k} \bar{\rho})\!\!\!\! \sum_{{s \colon a^{{i}}_{s} > 0}} \left(\delta_{sj} - \rho^{s} k^{{i}}_{s,{j}}\right).  $$

By setting $k^{{i}}_{s,{j}} = 0 \ \forall {j}$ when $a^{{i}}_{s} = 0$ (no attenuation along broken LORs through scattering voxels *s* geometrically incompatible with an SOR *i*), we simplify the sum over *s*: on the hull ($a^{{i}}_{{j}} > 0$), the expression determining the sign of (44) is then:

46 $$  1 - \sum_{s} \rho^{s} k^{{i}}_{s,{j}} \quad\Longleftrightarrow\quad \vec{1}_{[{i}]} \otimes \vec{1}_{[t]} - \vec{\rho}^{\top} \underline{\boldsymbol{K}},  $$

the multidimensional (size of a system matrix) analog of 1−*kρ*.

Note that $\underline {\boldsymbol {K}} \vec {\rho }$ (or $\sum _{t} k^{i}_{{j},t} \rho ^{t}$; used in (2) and the attenuation-factor expressions of (11 and 12)) is a matrix of radiological paths, so in terms of units of measurement, so is $\vec {\rho }^{\top } \underline {\boldsymbol {K}}$ (or $\sum _{s} \rho ^{s} k^{i}_{s,{j}}$; used in (11) and, in the following, (20)). However, there are fundamental differences: the former is the weighted sum of the red voxels in Fig. 15a [over all voxels *t* that attenuate for one specific broken LOR (*i*,*s*), weighted by *ρ*^*t*^], and as such,is the line integral of the (discrete) attenuation coefficient along the broken LOR. By contrast, the latter has a more complex geometrical interpretation (see Fig. 15b, bottom): it is the sum of the attenuating contributions of the *same* voxel *t* to all possible broken LORs (*i*,*s*), weighted by *ρ*^*s*^, the electron density in each broken LOR’s scattering voxel *s*. Low attenuation, in this case, corresponds to a small number of scattering voxels which are impacted by one attenuating voxel *j*, and thus a small overlap between scattering (green) and attenuating (red, blue) voxels across all broken LORs of an SOR.

In the minimal overlap case depicted in Fig. 15a, a voxel *j* on the football’s surface can have attenuating contributions to one of only three locations of each broken LOR (*i*,*s*), namely at *d*_*s*_, *s*, and *d*_*n*_ (where *d*_*s*_ and *d*_*n*_ will be outside of the unknown object). However, significant additional overlap may be the result of a number of factors: SORs with low curvature, that is, small scattering angle *θ* and large SOR radius *R*=*b*/(2 sin*θ*); scattering points *s* close to *d*_*s*_ or *d*_*n*_; consideration of energy uncertainty in ***A***_*λ*_; large subjects; and nonrectangular grids and large voxel sizes. An extreme example is a barely scattered coincidence (*E*≈511 keV,*θ*≈0,*R*→*∞*) on an LOR-like SOR *i*, along which, when $a^{i}_{j} > 0$, we find that $a^{{i}}_{s} > 0$ implies $k^{i}_{s,{j}} > 0$. So each voxel *j* attenuates (with its full effective length, $k^{{i}}_{s,{j}} \approx k^{i}_{{j},{j}}$) contributions from every scattering voxel; in other words, contributions from any scattering voxel *s* are attenuated by a voxel *j*. In this case, (46) reads:

47 $$  1 - k^{i}_{{j},{j}}\!\!\! \sum_{{s \colon a^{i}_{s} > 0}} \rho^{s} = 1 - l^{i}_{{j},{j}}\!\!\! \sum_{{s \colon a^{{i}}_{s} > 0}} \mu^{s} = 1 - l^{i}_{{j},{j}} n_{i} \bar{\mu},  $$

where *n*_*i*_ is the number of object voxels part of SOR *i*. With $l^{i}_{{j},{j}}$ on the order of the voxel size and $\bar {\mu } \approx {0.1}/\text {cm}$ in water, (47) has the same sign as (10cm−*L*_*i*_), where *L*_*i*_ is the path length through the object. So for very tight SORs, the interpretations of $\vec {\rho }^{\top } \underline {\boldsymbol {K}}$ and $\underline {\boldsymbol {K}} \vec {\rho }$ are similar, and the SOR through a water-like body of more than 10-cm thickness has $\partial \bar {y}^{i} / \partial \rho ^{j} < 0$; this is where the linear approximation underlying MLEM-OSL fails. The situation is more complex for other SORs.

## Added value of combining nonscattered and scattered data

We focus on an artificial single-voxel/single-detector-pair problem, with the expected true and scattered data:

48 $$ \bar{z} = (u \lambda) \exp(-u \rho) \quad\text{and}\quad \bar{y} = (b \lambda) \exp(-k \rho) \rho,  $$

respectively (compare (4) and (2) for multi-voxel variants). For a set of (*λ*,*ρ*) candidates, we plot the log-likelihood of trues and scatter as well as the joint log-likelihood, using system values *b*=*k*=*u*=1 and true values *λ*^true^=1 and *ρ*^true^∈{0.2,2} (low and high attenuation, respectively), chosen such that 0.2 exp(− 0.2)≈0.16 is in the range of 2 exp(− 2)≈0.27.

Figure 16 illustrates the added value of combining nonscattered (true) and scattered data, as compared to using only trues (MLAA) or only scatter (MLGA and scatter-MLEM). In both the low and the high attenuation example, the likelihood of trues exhibits the well-known scaling issue that can be appreciated in the form of an extended nonunique maximum; a similar effect is seen with the likelihood of scatter^9^. In this single-voxel example, the likelihood of scatter exhibits an additional nonuniqueness: for each value of *λ*, two values of *ρ* yield the same scatter likelihood, in line with two solutions of the 1-D scatter measurement equation (see Fig. 2); however, this nonuniqueness is more difficult to characterize with multiple voxels.

The intersection of the curves that follow the maxima features a unique intersection, which coincides with the maximum of the joint likelihood of trues and scatter data. The peak around this maximum is broader for high attenuation, in line with the lower angle of intersection between the maxima of the individual likelihoods.

## Data Availability

The datasets used during the current study are available from the corresponding author on reasonable request.

## Footnotes

1 SORs are single-scatter analogs of LORs, which comprise potential *emission* locations of *nonscattered* coincidences.

2 While *energy bin* may be a more appropriate term for list-mode data with energy information, and *energy window* more appropriate for list-mode data without energy information or histogrammed data, we use these terms interchangeably as we are only concerned with the maximum granularity of the energy information that can be obtained from the data.

3 This update using both terms is also responsible for vastly improved performance of MLGA compared to 2BP [24]: the full gradient of the data (11) is effectively a weighted sum of the 2BP update branches, where the weights ensure maximization of the log-likelihood.

4 The use of an elliptical phantom with relatively short, 12 and 15 cm long axes may have prevented this behavior from manifesting in an earlier study [18].

5 By contrast, downsampling or downsizing activity reconstruction is possible without such considerations.

6 This spatial scaling is identical, up to a global factor in the measurement data, to scaling the *μ*-map intensities by the same factor while maintaining all spatial dimensions—hence the interpretation in terms of high and low attenuation.

7 Note that neither the true solution nor the limit distribution are radially symmetric; the existence of nonradial nonuniqueness in 2D non-TOF PET has recently been proven [38] and may represent a practical problem.

8 This segmentation of the image space offers a geometric interpretation of the different back-projection kernels in the two branches of the 2BP update ([24], Eq. 17): the positive branch distributes an excess of measured coincidences (compared to expected ones) on SOR *i* by *increasing* *ρ* on the surface of the football (in voxels *j* where $\smash {a^{{i}}_{{j}} > 0 }$) and by decreasing *ρ* there for a lack of measured coincidences. By contrast, the negative branch *decreases* *ρ* (and hence *μ*) on the inside of the football (where $\smash {\sum _{s} k^{{i}}_{s,{j}} > 0 }$) for an excess of measured coincidences, and vice versa.

9 Note that the scatter log-likelihoods are different between low and high attenuation; they appear similar to each other due to the choice of *ρ*^true^ values.
